# Transcriptome Profiling in Rat Inbred Strains and Experimental Cross Reveals Discrepant Genetic Architecture of Genome-Wide Gene Expression

**DOI:** 10.1534/g3.116.033274

**Published:** 2016-09-19

**Authors:** Pamela J. Kaisaki, Georg W. Otto, Karène Argoud, Stephan C. Collins, Robert H. Wallis, Steven P. Wilder, Anthony C. Y. Yau, Christophe Hue, Sophie Calderari, Marie-Thérèse Bihoreau, Jean-Baptiste Cazier, Richard Mott, Dominique Gauguier

**Affiliations:** * The Wellcome Trust Centre for Human Genetics, University of Oxford, OX3 7BN, United Kingdom; † Cordeliers Research Centre, Institut National de la Santé et de la Recherche Médicale Unités Mixtes de Recherche Scientifique 1138, Sorbonne Universities, University Pierre & Marie Curie, University Paris Descartes, Sorbonne Paris Cité, 75006, France; ‡ Centre for Computational Biology, University of Birmingham, Edgbaston B15 2TT, United Kingdom; § University College London Genetics Institute, WC1E 6BT, United Kingdom

**Keywords:** epistasis, Goto–Kakizaki, diabetes mellitus, quantitative trait locus, eQTL

## Abstract

To test the impact of genetic heterogeneity on *cis*- and *trans*-mediated mechanisms of gene expression regulation, we profiled the transcriptome of adipose tissue in 20 inbred congenic strains derived from diabetic Goto–Kakizaki (GK) rats and Brown–Norway (BN) controls, which contain well-defined blocks (1–183 Mb) of genetic polymorphisms, and in 123 genetically heterogeneous rats of an (GK × BN)F2 offspring. Within each congenic we identified 73–1351 differentially expressed genes (DEGs), only 7.7% of which mapped within the congenic blocks, and which may be regulated in *cis*. The remainder localized outside the blocks, and therefore must be regulated in *trans*. Most *trans*-regulated genes exhibited approximately twofold expression changes, consistent with monoallelic expression. Altered biological pathways were replicated between congenic strains sharing blocks of genetic polymorphisms, but polymorphisms at different loci also had redundant effects on transcription of common distant genes and pathways. We mapped 2735 expression quantitative trait loci (eQTL) in the F2 cross, including 26% predominantly *cis*-regulated genes, which validated DEGs in congenic strains. A hotspot of >300 eQTL in a 10 cM region of chromosome 1 was enriched in DEGs in a congenic strain. However, many DEGs among GK, BN and congenic strains did not replicate as eQTL in F2 hybrids, demonstrating distinct mechanisms of gene expression when alleles segregate in an outbred population or are fixed homozygous across the entire genome or in short genomic regions. Our analysis provides conceptual advances in our understanding of the complex architecture of genome expression and pathway regulation, and suggests a prominent impact of epistasis and monoallelic expression on gene transcription.

The analysis of expression quantitative trait loci (eQTL) can provide novel insights into the function of disease-associated genes (Chen *et al.* 2008; Monti *et al.* 2008; Petretto *et al.* 2008; Heinig *et al.* 2010) and gene pathways and networks (Ghazalpour *et al.* 2006; Chen *et al.* 2008; Emilsson *et al.* 2008; Monti *et al.* 2008). The regulation of gene expression is orchestrated through complex local (*cis*-mediated) and distant (mediated *in trans*) mechanisms. eQTL studies in humans, which until recently were limited to cell systems, whole blood, and biopsies from the most accessible organs (Dixon *et al.* 2007; Emilsson *et al.* 2008; Fairfax *et al.* 2012; Grundberg *et al.* 2012), have now been scaled up to genetic analysis of transcriptome regulation across a broad range of tissues of many healthy individuals (GTExConsortium 2015). A key question is whether the predominantly *cis*-acting genetic architecture of gene regulation observed in studies of outbred populations, such as humans and heterogeneous stocks (Huang *et al.* 2009), is the complete picture. These study designs are underpowered to detect *trans*-regulation, yet the reproducibility of gene expression is governed, in large part, by the extent to which it is controlled *in cis* as opposed to *trans*. Therefore experimental designs in which the genetic control of gene expression is forced to be *in trans* can reveal aspects of regulation that are normally hidden. The use of congenics, in which genetic variation is confined to specific genomic segments but variation in gene expression can be measured genome wide, is key to understanding *trans* effects.

eQTL experiments in animal models can contribute to improving knowledge of eQTL architecture and elucidating the function of disease susceptibility loci identified in genome-wide association studies (Williams and Auwerx 2015). A broad range of experimental mammalian systems developed in the laboratory mouse (Buchner and Nadeau 2015) and rat (Gauguier 2005) allow the collection of organs from large cohorts of individuals maintained in strictly standardized conditions, thus limiting interindividual phenotype variability, and provide powerful tools for eQTL mapping. The inbred Goto–Kakizaki (GK) rat model of type 2 diabetes mellitus was produced over many generations of breeding rats from an outbred Wistar stock, using glucose intolerance as the sole criterion for selecting breeders (Goto *et al.* 1976). This process resulted in the isolation of the GK strain enriched for naturally occurring Wistar polymorphisms that contribute to diabetes and associated phenotypes, which we previously mapped by QTL analysis of pathophysiological phenotypes in F2 crosses between GK rats and Brown–Norway (BN) controls (Gauguier *et al.* 1996; Argoud *et al.* 2006). Further physiological phenotyping and multitissue transcriptome profiling in a congenic strain designed to contain a large (∼100 Mb) QTL-rich region of the GK rat in a BN background validated QTL effects and suggested that congenics can be efficiently used to dissect out *cis*- and *trans*-mediated regulation of gene transcription (Wallis *et al.* 2008).

Here we establish fundamental aspects of eQTL architecture and biology in adipose tissue, using three distinct but interrelated genetic settings, namely (i) inbred GK and BN strains, (ii) a panel of 20 congenic strains that carry well-defined blocks of GK and BN haplotypes across a total of 35% of the rat genome, and (iii) a large GK×BN F2 cross, in which we tested congenic transcriptome results. Transcriptome profiling (Illumina bead arrays) was used to detect differentially expressed genes and pathways between congenics and control animals, and to map *cis*- and *trans*-regulated eQTLs and eQTL hotspots in the cross. In the F2 cross, we were able to replicate only part of altered transcription regulation of genes and biological pathways detected in congenics. However, we show that the same biological pathways can be regulated by independent genomic loci, suggesting redundant biological function of distinct gene sets. Differential transcriptional regulation in inbred strains and segregating populations supports the important role of gene × gene interaction (*i.e.*, epistasis) in the control of genome expression.

## Materials and Methods

### Animals

**Figure 1:**
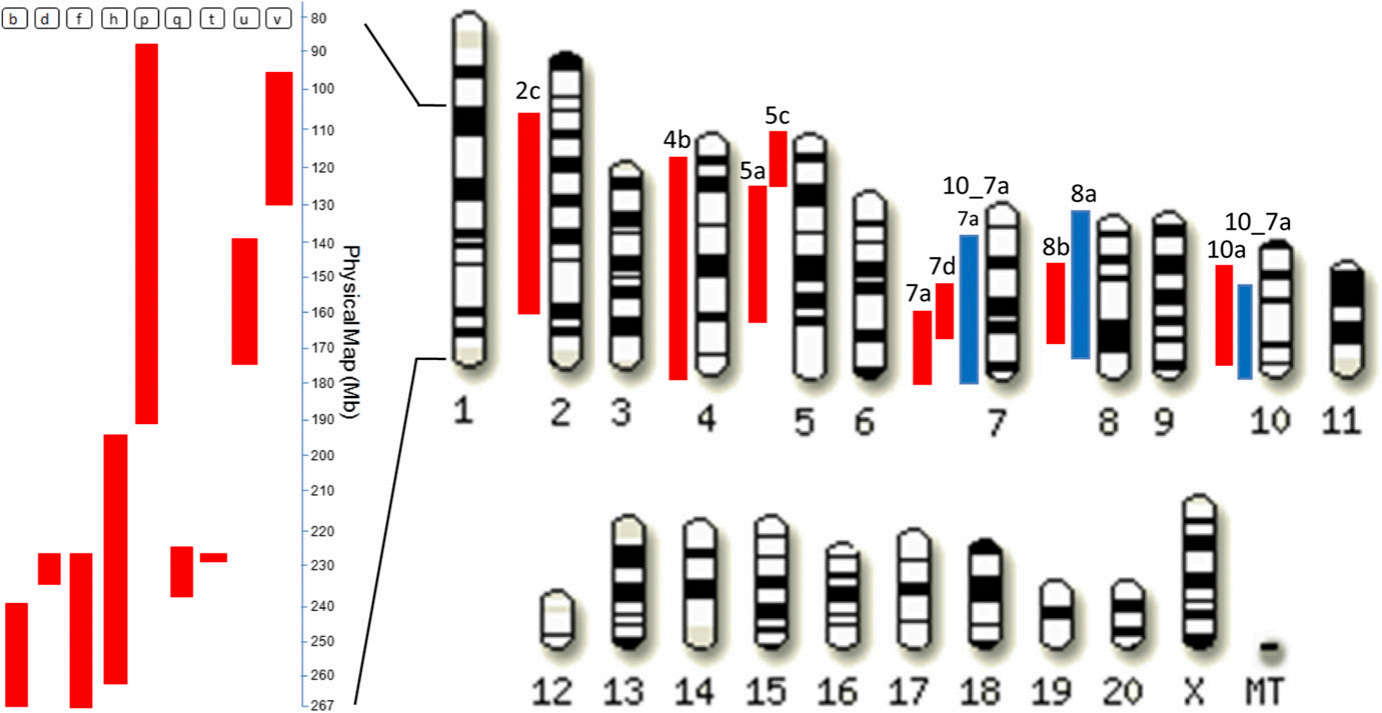
Schematic representation of the exchanged genomic regions in congenic strains. Red bars show the GK genomic blocks introgressed onto the genomic background of the BN strain in BN.GK congenic series. Blue bars show the BN genomic blocks introgressed onto the genomic background of the GK strain in GK.BN congenic strains. Details of the genomic intervals targeted in each congenic strain are given in Table S1.

A colony of GK/Ox rats bred at Biomedical Service Unit, University of Oxford, since 1995 from a GK/Par stock and BN rats obtained from a commercial supplier (Charles River Laboratories, Margate, UK) were used to produce a series of 20 BN.GK and GK.BN congenic strains using a genetic marker-assisted breeding strategy (Wallis *et al.* 2008). BN.GK congenics were designed to contain GK single genomic blocks introgressed onto the genetic background of the BN strain, whereas the reciprocal GK.BN congenics contained BN genomic blocks transferred onto a GK genetic background (Figure 1, Supplemental Material, Table S1). All congenic rats were genotyped as previously described (Wallis *et al.* 2008) to monitor retention of donor alleles across the congenic interval and their elimination from the genetic background. Male GK, BN, and congenic rats were used in all experiments. The F2 cross (*n* = 123) between rats of the GK/Par colony and normoglycemic BN controls, previously derived to map QTL for glucose tolerance, insulin secretion, adiposity, and metabolomic variables (Gauguier *et al.* 1996; Dumas *et al.* 2007), was used for eQTL mapping. The cohort consisted of 60 males and 63 females from two reciprocal crosses of 55 F2 rats originating from a GK female and 68 F2 rats originating from a BN female. Rats were allowed free access to tap water and standard laboratory chow pellets (B&K Universal Ltd, Grimston, Hull, UK) and were maintained on a 12-hr light/dark cycle. At 6 months, animals were euthanized after an overnight fast and retroperitoneal fat pads (RFP) were rapidly dissected, snap frozen in liquid nitrogen, and stored at −80°. Animal procedures were carried out under UK Home Office licenses and approved by the ethical review panel of the University of Oxford.

### RNA preparation

Total RNA was isolated from 100 mg of frozen RFP using the RNeasy 96 Universal Tissue kit (Qiagen, Crawley, UK). Briefly, frozen tissue samples were transferred into cooled RNeasy 96 Universal Tissue plates, and homogenized in QIAzol Lysis Reagent using Qiagen’s Tissue Lyser. Total RNA was purified using a spin technology according to the manufacturer’s guidelines and eluted in 90 µl of RNase-free water. RNA concentrations were determined using a NanoDrop spectrophotometer and RNA integrity was assessed using an Agilent 2100 Bioanalyzer (Agilent Technologies, Waldbronn, Germany).

### Illumina Bead Array hybridization, scanning, and data processing

Gene transcription profiling of RFP from F2 hybrids and from rats of the GK, BN, and congenic strains (six biological replicates per strain) was performed using Sentrix BeadChip RatRef-12 whole-genome gene expression arrays (Illumina Inc., San Diego, CA), containing 22,523 oligonucleotide probes (replicated on average 30 times), allowing quantification of transcript levels for 21,910 genes. Biological replicates were individually hybridized to the arrays.

Double-stranded cDNA and purified biotin-labeled cRNA were synthesized from 300 ng high-quality total RNA using the Illumina TotalPrep RNA amplification kit (Ambion Inc., Austin, TX). cRNA concentrations were determined using a NanoDrop spectrophotometer, and cRNA quality and integrity were assessed using an Agilent 2100 Bioanalyzer (Agilent Technologies). Hybridizations onto the arrays were carried out using 750 ng of each biotinylated cRNA. BeadChip arrays were scanned on the Illumina Bead Array Reader (Illumina Inc.). Data were analyzed using the Illumina BeadStudio Application software before undergoing comprehensive statistical analysis. Particular attention was given to the following quality control parameters: 0 ≤ G sat ≤ 1; Green 95 Percentile (GP95) for consistency between arrays (around 2000); GP5 background level in range of low 100 or below.

Whole genome sequencing of the GK/Ox strain (Atanur *et al.* 2013) identified variants between GK and BN in 757 Illumina oligonucleotides (Table S2), which were then excluded prior to array data analysis to avoid detection of spurious gene expression changes due to differences in binding between probes and oligonucleotides (Alberts *et al.* 2007). We verified absence of differential expression between GK, BN, and relevant congenics for several such genes (Figure S1). We also withdrew probes that detected only background signal (*i.e.*, Illumina detection score <0.5 in >50% of samples). Microarray data processing was carried out using normexp background correction and quantile normalization (Shi *et al.* 2010).

Microarray experiments were compliant with Minimum Information About a Microarray Experiment standards for reporting microarray experiments, and both protocol details and raw data have been deposited in ArrayExpress (http://www.ebi.ac.uk/arrayexpress/) under the accession numbers E-MTAB-969 (F2 hybrids) and E-MTAB-1048 (BN, GK, and congenic strains).

### Genetic mapping of expression QTL in the F2 (GK × BN) cross

eQTL analysis was performed using the R-qtl software package (Broman *et al.* 2003). We used genetic maps constructed in the cross with a combination of microsatellites and single nucleotide polymorphism markers (Wilder *et al.* 2004). Genome scans were carried out using the Haley–Knott regression method (Haley and Knott 1992). To account for effects of sex and lineage on gene expression, we used sex and cross direction as additive covariates in our models (Solberg *et al.* 2004). To obtain a genome-wide significance threshold for each transcript, we conducted a permutation test with 1000 permutations. QTL with a genome-scan adjusted *P*-value <0.05 were retained as significant.

We used a regression model with sex and cross direction as additive covariants:

$$\left(H_{a}\right)y_{i}=\mu +\beta_{c}c_{i}+\beta_{s}s_{i}+\beta_{g}g_{i}+\varepsilon_{i},$$

with *β_c_c_i_* denoting the effect of the cross, *β_s_s_i_* denoting the effect of sex, and *β_g_g_i_* denoting the effect of the genotype.

### Statistical analysis of Illumina array data in the congenic strains

Differential gene expression in the congenics was assessed by comparing array data in each congenic strain to the relevant parental strain (GK for GK.BN congenics and BN for BN.GK congenics). A linear model was fitted using linear models for microarray data (LIMMA) accounting for effects of experimental batches. Genes were tested for statistical differential expression using a moderated *t*-statistic and a threshold of *P* < 0.05.

### Pathway analyses

To obtain functional categories that are enriched in the eQTL-controlled gene sets in the F2 cross, we used a hypergeometric test on gene ontology terms and KEGG pathways associated with these gene sets against the background of genes with detectable expression (Falcon and Gentleman 2007). For functional analysis of the congenic transcriptomes, we used gene set enrichment analysis (GSEA) (Subramanian *et al.* 2005). We detected pathways in the KEGG and reactome databases that are enriched among upregulated or downregulated genes (Kanehisa and Goto 2000; Matthews *et al.* 2009). We used the standalone java version of GSEA 2.07 on the *t*-statistics of differential expression with default parameters and *n* = 1000 permutations.

### Quantitative real-time PCR

Total RNA was treated with Turbo DNA-free DNase kit, for removal of genomic DNA (Ambion Inc.). First-strand cDNA synthesis was performed using Superscript III First-strand Synthesis Supermix for quantitative real-time PCR (Invitrogen, Paisley, UK). Assays were performed on a Rotor-Gene 3000 system (Corbett Research, Milton, UK) using the QuantiTect SYBR Green PCR kit (Qiagen). Analysis was performed using the standard curve method (Rotor-Gene Software 5.0.47; Corbett Research). This software allowed further verification that a single PCR product signal was quantified. Gene expression was normalized against the expression of either actin or acidic ribosomal phosphoprotein P0 (36B4). Experiments were performed in duplicate with samples prepared from six animals per group. Statistical significance was determined by the two-tailed independent sample *t*-test or univariate ANOVA, when testing more than two groups. Oligonucleotides designed to test gene expression are given in Table S3.

### Data availability

The authors state that all data necessary for confirming the conclusions presented in the article are represented fully within the article.

## Results

### Differential transcription regulation in adipose tissue in GK and BN strains

We initially surveyed genome-wide transcriptional patterns in adipose tissue of male GK and BN rats (*n* = 6 per strain). We identified a total of 1221 genes showing evidence of significant differential expression [false discovery rate (FDR) < 0.05] with an equivalent number of genes upregulated (514) and downregulated (707) in the GK (Table S4). The proportion of genes differentially expressed between strains was similar across chromosomes (between 3.1% of genes on chromosome 15 and 6.3% of genes on chromosome 10).

### Transcriptional control by genetic polymorphism blocks in congenic strains

To establish the effects of blocks of linked genetic polymorphisms on gene transcription, we repeated fat transcriptome profiling in a series of reciprocal BN.GK and GK.BN inbred congenic strains (Figure 1 and Table S4) using the same Illumina platform. The targeted genomic blocks in congenics collectively covered up to 950 Mb (35%) of the rat genome length and contained >9900 protein coding genes (33.6% of the rat genes). BN.GK congenic strains each contain contiguous GK alleles within genomic blocks ranging in length from 1 to 183 Mb from rat chromosomes 1 (10 congenic lines), 2 (one congenic line), 4 (one congenic line), 5 (two congenic lines), 7 (two congenic lines), 8 (one congenic line), and 10 (one congenic line), independently introgressed onto the genetic background of the BN strain. The reciprocal GK.BN strains contained BN genomic blocks of rat chromosomes 7 (one congenic line), 8 (one congenic line), and 10 (one congenic line) transferred onto a GK genetic background. Thus, within a given congenic line, any difference in expression of a gene from the control line (if it is due to genetic differences) must be caused by polymorphisms within that congenic’s variable block.

In pairwise comparisons between congenics and relevant GK or BN control, using six male rats per strain, we identified a total of 4302 differentially expressed genes at FDR < 0.05 per comparison, ranging from 73 (BN.GK7d) to >1000 (BN.GK1b, 1d, 1h, 1p, and 1u) (Table 1). Gene density in congenic intervals had no apparent impact on the number of differentially expressed genes between congenics and controls. For example, congenics BN.GK1b, 1d, 1p, and 1u have the highest numbers of differentially expressed genes yet carry GK genomic blocks containing few genes (24 in BN.GK1d) or many genes (1407 in BN.GK1p), suggesting that transcriptional regulation footprint is related to the function rather than number of genes. A relatively small proportion (7.7% on average) of differentially expressed genes wase localized within the GK congenic blocks themselves, and may correspond to *cis*-regulated gene expression (Table S4). The remainder, which mapped outside the congenic region, unambiguously involve direct or indirect transcription regulatory mechanisms mediated *in trans*, as illustrated in Figure S2.

**Table 1:** Overview of fat gene transcription profiling in congenic strains of the GK rat

					Replicated GK *vs.* BN		Replicated eQTL	
Congenic Name	Gene Density	Total DEGs	Transcriptional Footprint	DEGs in Congenic Intervals, %	*N*	%	*N* (%)	Replicated *cis*-eQTL
BN.GK1b	203 (128)	1257	6.20	12 (1.0)	120 (3)	9.5 (2.5)	179 (14)	6
BN.GK1d	24 (11)	1239	51.71	1 (0.1)	76 (0)	6.1 (0)	171 (14)	0
BN.GK1f	394 (224)	874	2.24	21 (2.3)	77 (7)	8.7 (9.1)	148 (17)	8
BN.GK1h	994 (620)	1039	1.05	46 (4.4)	92 (15)	8.8 (16.3)	176 (17)	23
BN.GK1p	1407 (812)	1351	0.96	58 (4.3)	132 (20)	9.7 (15.2)	268 (20)	155
BN.GK1q	90 (45)	947	10.57	2 (0.2)	54 (0)	5.7 (0)	148 (16)	2
BN.GK1t	9 (4)	471	52.56	0 (0)	30 (0)	6.3 (0)	88 (19)	0
BN.GK1u	541 (363)	1337	2.48	19 (1.3)	114 (9)	8.5 (7.9)	196 (15)	11
BN.GK1v	210 (49)	931	4.45	6 (0.6)	55 (2)	5.9 (3.6)	122 (13)	28
BN.GK2c	1243 (627)	101	0.08	27 (26.7)	33 (16)	32.7 (48.5)	36 (36)	28
BN.GK4b	1578 (812)	232	0.15	26 (11.1)	45 (13)	19.2 (28.9)	49 (21)	26
BN.GK5a	511 (275)	94	0.18	9 (9.6)	18 (6)	19.1 (33.3)	17 (18)	10
BN.GK5c	259 (112)	557	2.15	12 (2.2)	44 (6)	7.9 (13.6)	67 (12)	5
BN.GK7a	576 (345)	91	0.16	13 (14.3)	30 (8)	33.0 (26.7)	30 (33)	14
BN.GK7d	275 (151)	73	0.27	4 (5.5)	9 (2)	12.3 (22.2)	13 (18)	5
BN.GK8b	448 (259)	312	0.71	8 (2.5)	36 (5)	11.3 (13.9)	25 (8)	7
BN.GK10a	1432 (870)	294	0.21	38 (12.9)	34 (12)	11.6 (35.3)	50 (17)	25
GK.BN7a	1014 (580)	54	0.05	11 (20.4)	19 (6)	35.2 (31.6)	16 (30)	12
GK.BN8	1149 (675)	486	0.42	32 (6.6)	74 (12)	15.2 (16.2)	71 (15)	19
GK.BN10_7a	2166 (1276)	140	0.07	39 (27.7)	49 (22)	34.8 (44.9)	54 (38)	40

Gene density indicates the number of genes localized in the congenic interval and in parentheses those represented on the Illumina BeadChip. Transcriptome data were analyzed to identify differentially expressed genes (DEGs) (*P* < 0.05) in BN.GK and GK.BN congenic series when compared to BN and GK controls, respectively. The transcriptional regulatory footprint of each congenic region was calculated as the ratio of the total number of DEGs between congenics and control to the number of genes localized in the corresponding congenic interval. The number and percentage of DEGs localized in each congenic interval were determined. Replication of expression changes in parental strains and in congenics was evaluated by the number (*N*) and percentage of genes found consistently differentially expressed (same direction of expression change) between congenic and controls and between GK and BN, including those localized in congenic intervals (in parentheses). Replicated eQTL effects correspond to DEG between congenic and controls that are linked to eQTLs showing consistent direction of expression changes mediated by GK alleles in the F2 rats and in congenics. *Cis* eQTLs replicated in congenics are shown. Full details of individual DEG in inbred strains (GK, BN, and congenics) and eQTLs are given in Table S4

**Figure 2:**
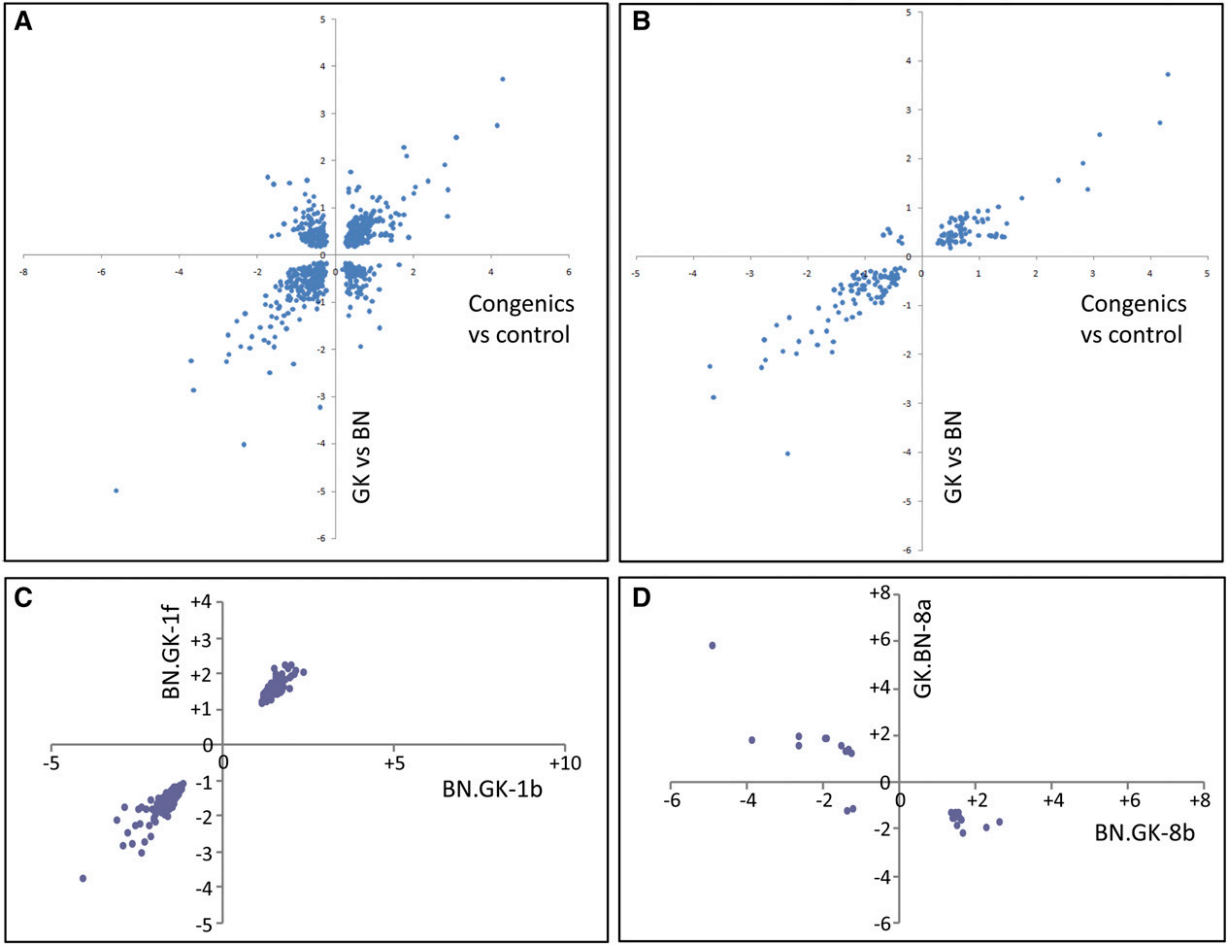
Patterns of gene transcription regulation in white adipose tissue by GK/BN polymorphisms in GK and BN rats and in congenic strains. Conserved gene expression patterns between BN.GK congenic rats and controls and between GK and BN rats are shown for all differentially expressed genes (A) and those localized in congenic intervals (B). Conservation of gene expression changes in congenics targeting overlapping genomic regions is illustrated by plotting expression ratio in strains BN.GK1b and BN.GK1f (C) and in reciprocal strains BN.GK8b and GK.BN8a (D). Full details of differentially expressed genes are given in Table S4.

To test the consistency of gene transcription patterns in congenic and control strains, we analyzed the set of 692 genes found significantly differentially expressed between congenic rats and their relevant BN or GK controls, and between GK and BN rats. Of these, 469 (68%) showed consistent direction of expression changes in the two comparisons (Table 1 and Figure 2A). Remarkably, when only genes mapped within congenic intervals were considered, we found almost complete concordance in directions (159 of 165; 96%) in the two comparisons (Figure 2B), suggesting conservation of *cis*-mediated control of gene transcription but a lack of conservation of *trans*-mediated control. The power to detect gene expression changes was independent of whether a gene was inside a congenic block.

To assess robustness of differential gene expression in congenic rats, we compared transcriptome data in congenic strains that contained overlapping genomic regions on chromosome 1. Expression patterns of genes mapped to shared GK genomic blocks in different congenics showed near complete consistency, as illustrated in Figure 2C for BN.GK1b and BN.GK1f. Data from reciprocal (BN.GK and GK.BN) congenics, which target the same >40 Mb exchanged genomic segments on chromosomes 7 (BN.GK7a, GK.BN7a, and GK.BN10_7a), 8 (BN.GK8b and GK.BN8a), and 10 (BN.GK10a and GK.BN10_7a) (Figure 1 and Table S1), also supported consistent (but reversed, because of the reciprocity of the congenics) gene expression patterns. They provide strong evidence of robust transcriptional effects of BN and GK alleles at these loci when expressed in contexts of GK and BN genome backgrounds, respectively. Thus, 24 of the 26 differentially expressed genes localized in the shared 40 Mb exchanged region in BN.GK8b and GK.BN8a showed opposite expression trends, demonstrating conserved allelic effects on gene transcription (Figure 2D).

**Figure 3:**
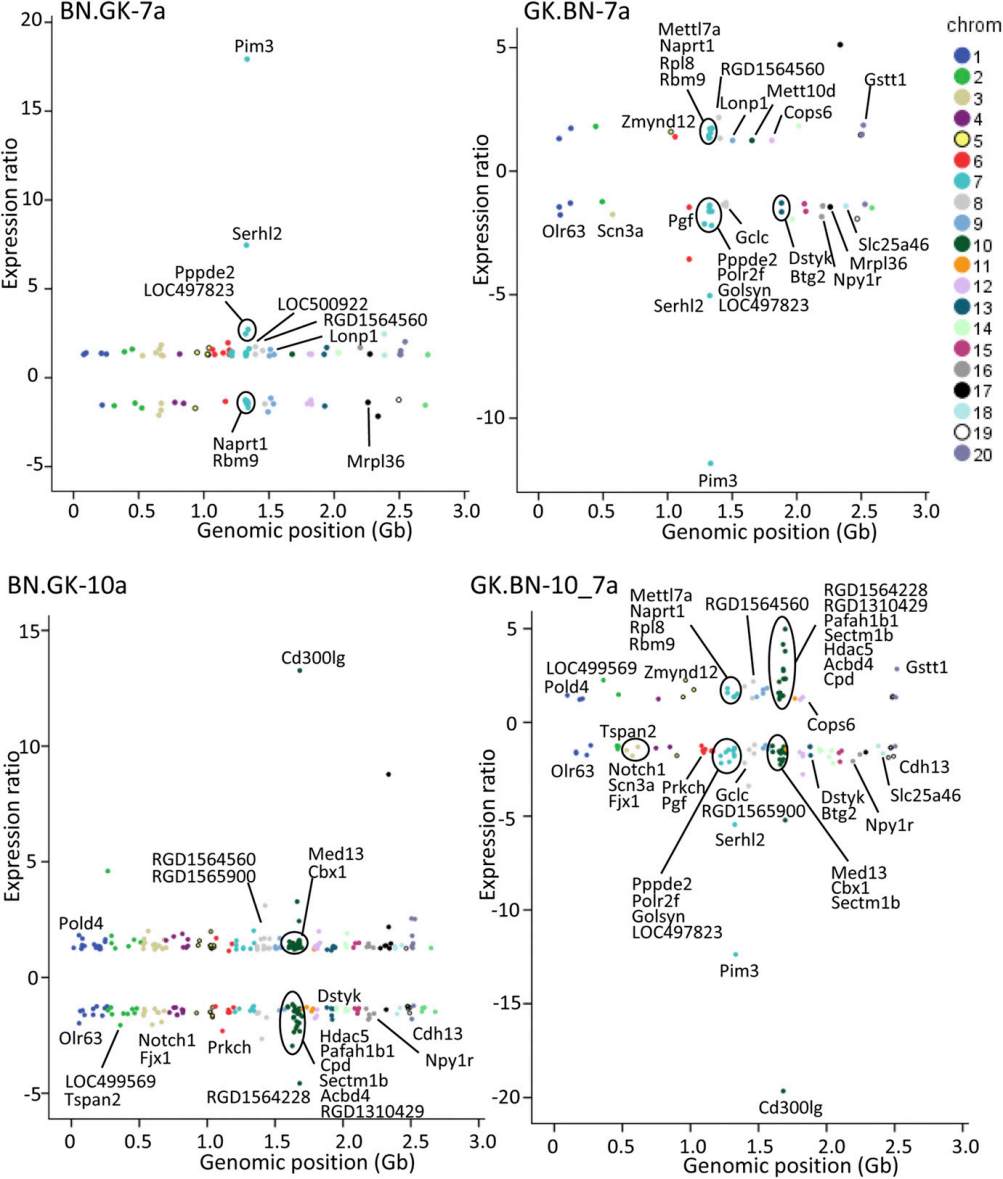
Patterns of gene transcription regulation in congenic strains. Conserved allelic effects on genome-wide gene expression regulation in adipose tissue were tested in BN.GK7a and BN.GK10a congenics and in reciprocal strains GK.BN7a and GK.BN10_7a targeting largely overlapping genomic regions. Genomic positions of genes significantly differentially expressed (*P* < 0.05) between BN.GK congenic and BN controls and between GK.BN congenic and GK controls are plotted along the *x*-axes and gene expression ratios are shown along the *y*-axes. Chromosomal location of genes is color coded. Full details of differentially expressed genes, including genes that are localized in the congenic intervals and may be regulated in *cis*, are given in Table S4.

These results were further corroborated in the double congenic GK.BN10_7a, which contains two BN genomic blocks of chromosomes 7 and 10 introgressed onto a GK background. It combines the same regions of chromosome 7 as BN.GK7a and GK.BN7a, and the same region of chromosome 10 as BN.GK10a (Figure 1 and Table S1). Gene transcription changes in GK.BN10_7a were remarkably consistent with BN.GK7a and GK.BN7a data for genes localized in chromosome 7 (*e.g.*, *Pim3*, *Serhl2*, *Naprt1*, and *Rbm9*) and BN.GK10a data for genes mapped to chromosome 10 (*e.g.*, *Cd300lg*, *Cpb*, *Med13*, and *Cbx*) (Figure 3 and Table S4). To a lesser extent, consistent transcriptional regulation in these congenics was also observed for genes mapped outside the exchanged genomic interval, demonstrating conserved *trans*-mediated gene expression patterns.

Importantly, the majority of those genes outside of the congenic block that are differentially expressed exhibit changes very close to twofold up or down (*i.e.*, +/−1 on the base-2 log scale in Figure 3 and Table S4), allowing for the statistical uncertainties in the estimated expression levels. In contrast, differentially expressed genes within the congenic block showed unconstrained changes. This suggests that the *trans* effects are largely confined to transitions between monoallelic and biallelic expression, and thus are both qualitatively and quantitatively distinct from the *cis* effects.

The congenic series thus provide a powerful experimental system to discriminate *cis*- and *trans*-mediated mechanisms of transcription of individual genes driven by polymorphisms present in specific genomic blocks.

### Genomic blocks in congenic strains regulate specific and redundant biological pathways

**Figure 4:**
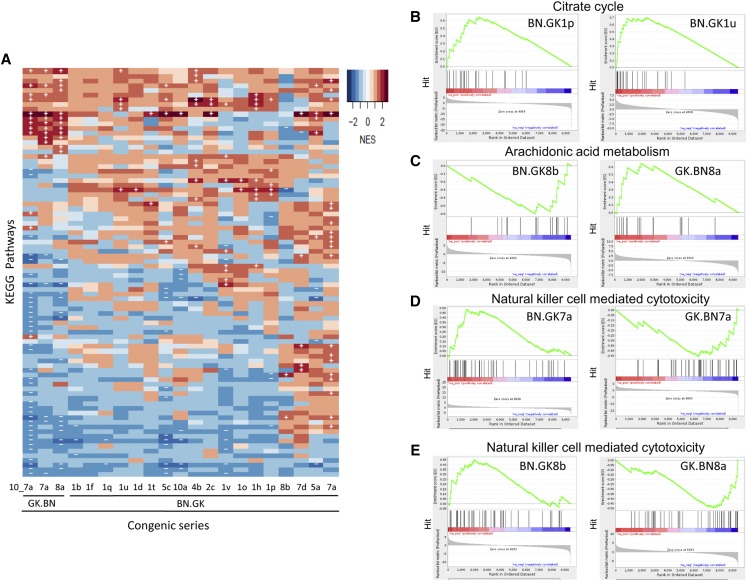
Global effects of GK/BN polymorphisms in congenic strains on biological pathways in white adipose tissue. Hierarchical clustering of differentially regulated pathways in congenic strains illustrate shared and strain specific biological mechanisms regulated by polymorphisms in genomic blocks (A). Pathways downregulated or upregulated in congenics are shown in blue and red cells, respectively. Statistically significant changes are indicated by − and +. Enrichment plots of genes contributing to significant alterations of biological pathways in congenics (B–E) illustrate conserved patterns of pathway regulation in congenics BN.GK1p and 1u (B) and contrasting patterns in reciprocal congenic strains (BN.GK and GK.BN) targeting overlapping regions of chromosomes 7 (D) and 8 (C, E). Output images of GSEA show the number of genes (vertical lines) upregulated (red bars) or downregulated (blue bars) contributing to overall stimulation or inhibition of the pathways (green curves). Full lists of altered KEGG pathways in congenics are shown in Table S5 and details of differentially expressed genes between congenics and controls are given in Table S4. Details of genes contributing to enrichment of pathways in B–E are shown in Table S7.

To evaluate the biological consequences of coordinated gene transcription changes in congenics, we carried out GSEA. We identified 87 KEGG and 100 reactome pathways altered in at least one congenic strain (Table S5 and Table S6). Analysis of KEGG pathways showed that the two reciprocal congenic series containing GK genomic blocks on a BN background (BN.GK) and BN genomic blocks on a GK background (GK.BN) can be separated, even though several pathways are similarly affected in these two congenic series (Figure 4A). Concordant KEGG pathway regulation was observed in congenics carrying overlapping GK genomic regions (Figure 4B and Table S5). For example, significant upregulation of the citrate cycle pathway, as defined by positive enrichment scores (ES) given by GSEA, was replicated in BN.GK1p (ES = 1.97; FDR *q*-value = 0.02) and 1u (ES = 2.14; FDR *q*-value = 3.2 × 10^−3^) (Figure 4B), and involved a common set of 12 genes (*Acly*, *Aco1*, *Aco2*, *Dlat*, *Dld*, *Dlst*, *Idh1*, *Idh3b*, *Pc*, *Sdha*, *Sdhc*, and *Sdhd*) (Table S7) that map outside the congenic intervals and contribute to pathway enrichment among the 20 genes tested in the pathway, suggesting a consistent effect of genetic polymorphisms localized in the shared GK genomic block on distant genes.

As observed at the gene level, contrasting patterns of pathway regulation were found in reciprocal BN.GK and GK.BN congenics, indicating coherent effects of GK and BN alleles in the targeted region, when transferred onto BN and GK genomic backgrounds, respectively. For example, pathways downregulated in GK.BN8a (arachidonic acid metabolism, natural killer cell-mediated cytotoxicity) and GK.BN7a (natural killer cell-mediated cytotoxicity) were upregulated in BN.GK8b and BN.GK7a (Figure 4, C–E). As noted above for the regulation of the citrate cycle in BN.GK1p and 1u, a core set of 6–7 genes contributed to pathway enrichment in reciprocal congenics, in addition to 4–15 strain-specific contributing genes (Table S7).

Results from pathway analysis also provide evidence of alteration of the same biological mechanisms in BN.GK strains containing different GK genomic regions. For example, the ribosome pathway was systematically upregulated in BN.GK2c (ES = 2.38; FDR *q*-value < 0.001), 5a (ES = 2.18; FDR *q*-value < 0.001), 5c (ES = 2.53; FDR *q*-value < 0.001), 7a (ES = 2.45; FDR *q*-value < 0.001), and 10a (ES = 2.37; FDR *q*-value < 0.001) (Table S5). Even though these strains carry different collections of GK variants (Figure 1), we identified a common set of 25 genes that contributed to pathway enrichment in all five congenics (Table S8), demonstrating *trans* effects of different regions converging on a common biological pathway. Along the same lines, consistent downregulation of natural killer cell-mediated cytotoxicity in GK.BN7a and GK.BN8a (Figure 4, D and E and Table S7), which target different chromosomes (Figure 1 and Table S1), involved a common set of 15 genes (*Bid*, *Cd48*, *Chp2*, *Fcgr3a*, *Fyn*, *Icam2*, *Klrd1*, *Lat*, *Lck*, *Map2k1*, *Nras*, *Plcg1*, *Prf1*, *Rac1*, and *Tnfsf10*) among the 40 genes of the pathway (Table S7). Interestingly, significant upregulation of this pathway in both BN.GK7a and BN.GK8b (Figure 4, D and E) confirmed the contribution of four genes (*Bid*, *Lat*, *Lck*, and *Prf1*), but involved a greater number of genes specific to these two BN.GK strains (*Hcst*, *Plcg2*, *Ppp3ca*, *Ppp3r1*, *Ptpn6*, *Rac2*, and *Vav1*) when compared to GK.BN strains (Table S7), suggesting interactions between alleles in the background and in the congenic intervals in the control of this pathway.

These results underline conserved and strain-specific functional consequences of genetic polymorphisms located in specific genomic blocks. They also demonstrate that distinct genomic regions have functionally redundant roles on biological pathways, which may be regulated by different series of genetic polymorphisms or involve epistasis.

### Genetic architecture of fat gene transcription regulation in (GK × BN) F2 hybrids

**Figure 5:**
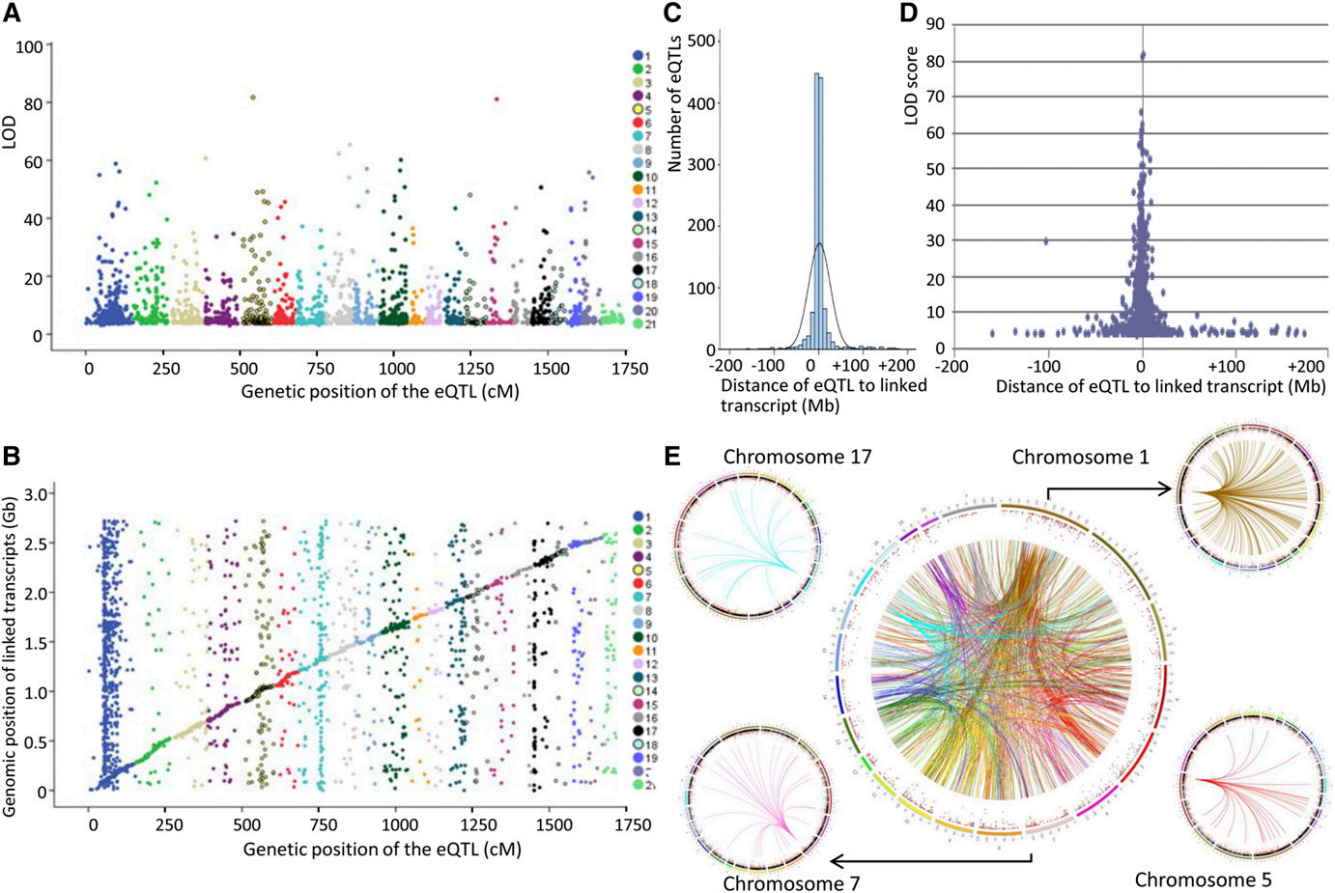
Overview of eQTL features in adipose tissue of GK × BN F2 hybrids. Genetic positions of statistically significant eQTLs are plotted against the LOD scores (A). Local and distant eQTLs are illustrated by plotting genetic positions of statistically significant eQTLs and the genomic position of the linked transcripts (B). Data from pairs of eQTL and linked transcripts mapped to the same chromosomes were used to determine the distribution of genomic distances between transcripts and genetic markers showing the strongest evidence of statistically significant linkage in the cross (C) and relationships with the significance of genetic linkages (D). Data from pairs of transcripts and eQTLs mapped to different chromosomes were selected to illustrate distant (*trans*) effects of genetic loci on gene transcription and *trans*-acting eQTL hotspots (smaller circles) (E). Chromosomes are color coded on the circle. The colors of the lines illustrate the effects of eQTLs mapped to the same chromosomes on the expression of distant genes. Details of eQTLs are given in Table S4. LOD, Logarithm of the odds.

To validate architectural features of gene transcription identified in the GK rat and in congenic strains, we mapped eQTLs in white adipose tissue of rats from a GK × BN F2 cross. Genotypes at over 255 framework markers typed in the cross were used to impute allele probabilities and construct a map of 898 marker positions (2.5 cM spacing between markers). Following withdrawal of oligonucleotides containing DNA variants, markers were tested for linkage to 15,822 detectable Illumina array signals in each of the 123 F2 rats. We identified a total of 2735 eQTLs at FDR < 0.05, including 2483 (90.8%) linked to transcripts corresponding to genes unambiguously localized in the rat genome assembly (RGSC3.4, Ensembl release 69) (Figure 5A and Table S9).

Results from transcriptome analyses in the F2 showed classical features of eQTL architecture, including local (*cis*) and distant (*trans*) genetic control and eQTL hotspots. A total of 1500 eQTLs were linked to transcripts mapped to different chromosomes, which unequivocally indicated the involvement of *trans*-mediated regulation of gene transcription (Figure 5B and Table S4). A high proportion of the remaining 1159 eQTLs located to the same chromosomes as the linked transcripts were localized within 5 Mb (*n* = 698; 60%), 10 Mb (*n* = 910; 79%), or 20 Mb (*n* = 1033; 89%) of the linked transcripts, strongly suggesting *cis*-mediated transcription regulation (Figure 5C and Table S4). The most statistically significant of these eQTLs were localized in close vicinity to the linked transcripts (Figure 5D). eQTLs were evenly distributed across the genome, with an average of 4.8% of eQTLs per chromosome (1 Mb average spacing between eQTLs), with the exception of chromosome 1 which showed a large excess (27.1%) of *trans*-regulating eQTLs (Figure 5B and Table S9), even though it only covers ∼10% of the rat genome length and only contains 13% of rat genes. This eQTL excess can be explained by a hotspot of 319 eQTLs in a short region of chromosome 1 (49.4–59.4 cM, 90.3–96.9 Mb) (Figure 5E and Table S4). Other eQTL hotspots were detected on chromosomes 5 (57.5–62.5 cM, 138.3–146.4 Mb), 7 (70.0–72.5 cM, 113.0–117.0 Mb), and 17 (6.1–8.6 cM, 11.4–14.6 Mb). GK alleles at the eQTL hotspots on chromosomes 1, 7, and 17 were associated with downregulated expression of a high proportion of distant genes (64–73%), suggesting that eQTL clustering may be biologically relevant and reflect genuine mechanisms of gene transcription control driven by the eQTLs.

### eQTLs in outbred F2 hybrids partially replicate transcriptome architecture in inbred strains

**Figure 6:**
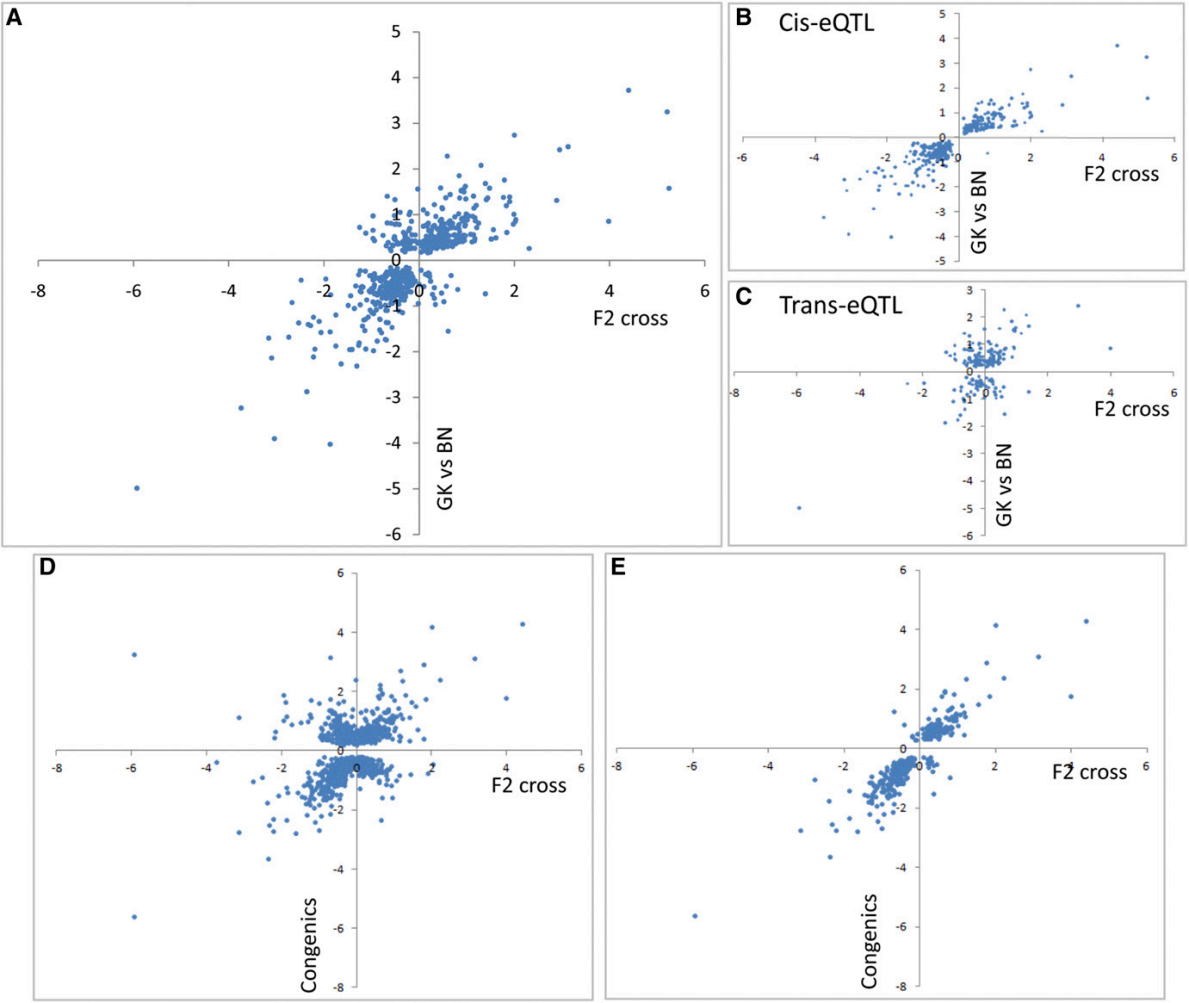
Correlation analysis of the effects of GK alleles on genome-wide gene expression in F2 hybrids and in inbred strains. The effects of GK alleles at statistically significant eQTLs (*P*-adjusted < 0.05) in the GK × BN F2 cross are plotted against expression ratio of corresponding genes significantly differential expressed (*P*-adjusted < 0.05) between GK and BN strains for all eQTLs (A) and *cis*- (B) and *trans*- (C) mediated eQTLs. The effects of GK alleles at eQTLs on gene expression regulation were compared in the F2 cross and in congenic strains for genome-wide gene expression data (D) and for genes mapped to congenic intervals (E). Details of differentially expressed genes between inbred strains and gene expression at eQTLs are given in Table S4.

To assess the relevance of eQTLs to genome-wide transcription regulation in inbred strains, we initially compared gene differential expression between GK and BN with transcriptional effects of corresponding eQTLs in the cross. Only 50% of differentially expressed genes between these strains (614 of 1221) corresponded to eQTLs. Even though this only represents a small proportion (22%) of eQTLs, the effect of GK alleles on the direction of gene expression changes was consistent for the majority (86%; *n* = 531) of these (Figure 6A). In addition, allelic effects on gene transcription in the F2 rats and inbred strains were consistent for all 362 *cis*-regulated eQTLs (Figure 6B) but for only 58% of *trans*-mediated eQTLs (Figure 6C).

We then compared genome-wide gene expression data in congenics and in F2 hybrids. The existence of several eQTLs was verified by transcription analysis of the linked genes in congenics and controls (Figure S1). We identified 1188 genes differentially expressed between congenics and controls that were linked to eQTLs, including 708 (60%) with concordant effects of GK alleles on the direction of gene expression changes in F2 and congenics (Figure 6D and Table S4). The resulting 26% replication rate in the F2 cross of differential gene expression in congenics is consistent with gene density in the congenic intervals (33.6% of all rat genes). When only eQTLs for the 358 differentially expressed genes localized in congenic intervals were considered, allelic effects on the direction of gene expression change in F2 and in congenics were consistent in the vast majority of cases (*n* = 335; 94%) (Figure 6E and Table S4). Of note, both *cis*- and *trans*-acting eQTLs were equally validated in congenics when the corresponding genes were localized within the genomic blocks of the congenic strains (Figure S3).

To investigate *trans*-regulated eQTLs in more detail, we tested if the F2 eQTL hotspot detected in chromosome 1 was replicated in the congenic strain BN.GK1p, which contains GK alleles in this region. Over 36% (98 of 270) of *trans*-regulated genes in this eQTL hotspot were significantly differentially expressed between BN.GK1p and BN (Table S10). Remarkably, for all 98 genes, the effect of GK alleles on the direction of gene expression changes was consistent in F2 hybrids and congenics. Congenic strains BN.GK1u and BN.GK1v, which share large GK haplotypes with BN.GK1p but are distal to this eQTL hotspot, showed more limited differential expression of eQTL genes (14 and 8%, respectively). These data confirm the existence of this *trans*-eQTL hotspot driven by regulatory elements in the GK region specific to BN.GK1p.

### Congenic transcriptome pathways do not predict F2 eQTL biological function

To test replication of biological features of fat transcriptomes identified in GK congenics, we repeated pathway enrichment analysis in the F2. Significantly affected pathways in the F2 included diabetes mellitus, digestion and absorption of fat, immunological processes (phagosome, allograft rejection, antigen processing and presentation, autoimmune diseases, cell adhesion molecules, natural killer cell-mediated cytotoxicity, and hematopoietic cell lineage), and metabolism of fatty acids and 11 amino acids (Table 2). Given the importance of the GK rat as a model for diabetes, this suggests that many GK/BN alleles across the genome, and that segregate in the cross, contribute to the metabolic and inflammatory mechanisms described in human diabetes.

**Table 2:** KEGG pathways underlying eQTL biological effects in the white adipose tissue transcriptome in the GK × BN F2 cross

						Congenics	
Rank	KEGG ID	*P* Value	Count	Size	Term	Upregulation	Downregulation
1	5332	<0.01	17	34	Graft *vs.* host disease	—	—
2	4145	<0.01	41	128	Phagosome	—	—
3	4940	<0.01	18	39	Type 1 diabetes mellitus	—	—
4	5416	<0.01	24	61	Viral myocarditis	—	GK.BN7a, 8a, 10_7a
5	4612	<0.01	22	58	Antigen processing and presentation	BN.GK7a	BN.GK1p; GK.BN10_7a
6	5330	<0.01	16	37	Allograft rejection	—	—
7	5320	<0.01	16	39	Autoimmune thyroid disease	—	—
8	4514	<0.01	29	92	Cell adhesion molecules	—	BN.GK1v,10a; GK.BN8a, 10_7a
9	1100	<0.01	167	798	Metabolic pathways	—	—
10	5140	<0.01	19	53	Leishmaniasis	BN.GK7a, 7d	GK.BN10_7a
11	5322	<0.01	19	59	Systemic lupus erythematosus	—	—
12	380	<0.01	11	27	Tryptophan metabolism	—	—
13	5150	0.01	12	33	*Staphylococcus aureus* infection	—	—
14	250	0.01	9	22	Alanine, aspartate, and glutamate metabolism	—	—
15	280	0.01	12	34	Valine, leucine, and isoleucine degradation	BN.GK5a; GK.BN7a, 8a, 10_7a	BN.GK7d
16	360	0.01	5	9	Phenylalanine metabolism	—	—
17	350	0.01	7	16	Tyrosine metabolism	—	—
18	650	0.02	7	17	Butanoate metabolism	—	—
19	71	0.02	11	33	Fatty acid metabolism	GK.BN7a, 8a	—
20	790	0.02	4	7	Folate biosynthesis	—	—
21	5144	0.02	11	34	Malaria	—	—
22	511	0.02	6	14	Other glycan degradation	—	—
23	4650	0.02	19	70	Natural killer cell-mediated cytotoxicity	BN.GK7a, 8b	GK.BN7a, 8a, 10_7a
24	4640	0.02	15	52	Hematopoietic cell lineage	—	GK.BN10_7a
25	982	0.03	13	44	Drug metabolism – cytochrome P450	GK.BN7a, 8a, 10_7a	—
26	270	0.03	9	27	Cysteine and methionine metabolism	BN.GK1v; GK.BN7a	—
27	4975	0.03	9	27	Fat digestion and absorption	—	—
28	450	0.04	5	12	Selenocompound metabolism	—	—
29	4970	0.05	15	57	Salivary secretion	—	—
30	4512	0.05	15	57	Extra-cellular matrix-receptor interaction	—	BN.GK1b, 1q, 1v, 5c, 10a; GK.BN8a, 10_7a

Data were analyzed with sex and cross as additive covariates. Congenic strains showing upregulation or downregulation in pathways identified in F2 hybrids are listed. Details of pathways can be found at www.genome.jp/kegg/.

The vast majority of pathways found altered in congenics, which may be directly or indirectly caused by genetic polymorphisms present in the congenic intervals, were not validated in F2 hybrids (Table S5), suggesting that the biological effects of genetic polymorphisms isolated in contiguous genomic blocks in inbred congenics may be counter-regulated by gene × gene interactions, when alleles across the genome segregate in outbred individuals.

## Discussion

We report the comprehensive genetic analysis of genome-wide fat gene transcription in a series of inbred congenic strains followed by replication in genetically heterogeneous F2 hybrids derived from the same combination of rat strains. We demonstrate conserved and discordant transcript regulation when genetic polymorphisms across the entire genome are fixed homozygous or segregate in a cross, and when they are dissected out in genomic blocks in congenic strains. Comparative analyses of transcriptomes in these systems provide an original experimental framework to assess the extent and impact of epistasis on the regulation of gene expression and biological pathways.

Transcriptome regulation in GK × BN F2 hybrids exhibits typical architectural features of eQTLs described in genetically heterogeneous populations in humans (Dixon *et al.* 2007; Emilsson *et al.* 2008; Grundberg *et al.* 2012; GTExConsortium 2015) and in mapping panels in mice (Schadt *et al.* 2003; Bystrykh *et al.* 2005; Chesler *et al.* 2005) and rats (Hubner *et al.* 2005; Petretto *et al.* 2006), including *cis*- and *trans*-mediated eQTLs and eQTL clusters controlling the transcription of large numbers of distant genes (Schadt *et al.* 2003; Emilsson *et al.* 2008). The definition of *cis*-eQTLs and eQTL hotspots in these genetic contexts is based on arbitrary estimates of physical distances separating genetic markers and linked transcripts (Petretto *et al.* 2006). Also, the existence of eQTL hotspots has been questioned (Breitling *et al.* 2008) due to the low effect size of *trans*-regulated eQTLs (Zhu *et al.* 2008). Assessment of these features in F2 cohorts is complicated by extensive linkage disequilibrium, preventing the high resolution mapping needed to separate closely linked eQTLs.

In contrast, the genetic structure of congenic strains allows for a cruder measurement of gene differential expression caused by exchanging genetic polymorphisms present in well-defined genomic intervals in an otherwise uniform background, thereby unambiguously distinguishing between *cis*- and *trans*-mediated regulations (Buchner and Nadeau 2015). Differential expression of genes mapped within the genomic regions exchanged in congenics should therefore correspond to *cis*-regulated effects whereas differential expression of genes mapped outside these regions unambiguously indicates *trans*-mediated regulation. Perhaps surprisingly, we find that almost all *trans*-regulation in the congenics takes the form of twofold changes in expression, which we interpret as transitions between monoallelic and biallelic expression. This is reminiscent of expression changes observed in reciprocal crosses of mouse gene knockouts (Chess 2012; Mott *et al.* 2014), and suggests a different mechanism for *trans* regulation compared to *cis*. This simple genetic architecture is not observed in the F2 cross, presumably because many *trans* loci regulate a given gene, smoothing out the individual effects that only become evident in the congenics. We suggest that *trans* effects in outbred populations, including humans, are accumulations of multiple additive and nonadditive (epistatic) combinations of switches. This hypothesis deserves further investigation; for example, by building a series of combined GK.BN congenics in which the fraction of the genome that is GK accumulates in a known manner. Similarly, analysis of F1 hybrids of congenics (to generate heterozygosity) would demonstrate if dominance effects mask the effects observed in pure congenics.

**Figure 7:**
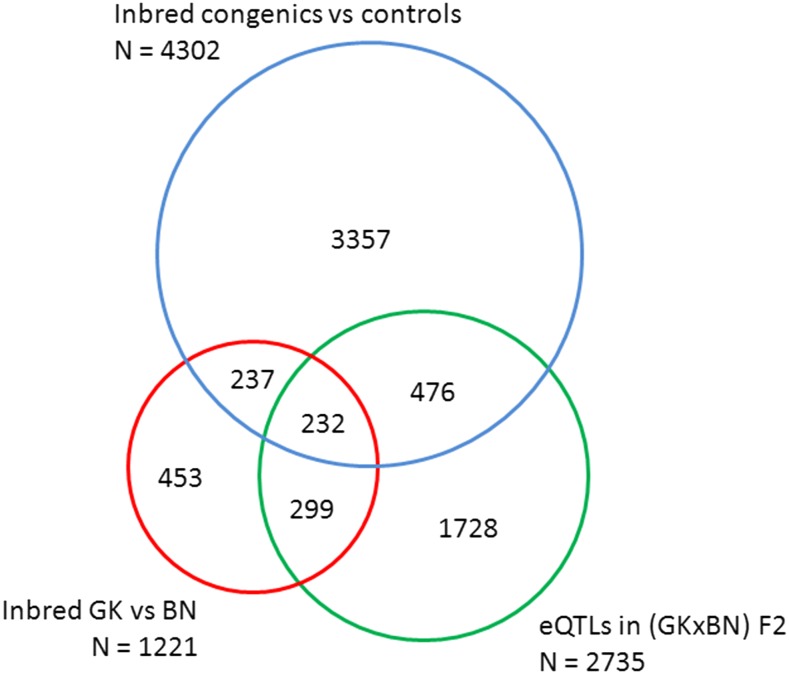
Summary of transcriptome results among inbred strains and eQTL data in GK × BN F2 hybrids. The number of statistically significant eQTLs (green circle) and differentially expressed genes between GK and BN (red circle) and between congenics and controls (blue circle) are reported. Concordant data between the different experimental groups correspond to genes showing consistent allelic effects on the direction of expression changes.

Congenic strains targeting overlapping genomic regions showed remarkably consistent patterns of gene transcription, and we found predominantly *cis*-regulated eQTLs and an eQTL hotspot in congenics, despite the 8-yr time-lag required to produce the congenic rats after the F2 hybrids (Gauguier *et al.* 1996; Wallace *et al.* 2004; Wallis *et al.* 2004). Although the congenic strains collectively target ∼30% of the rat genome, the total number of differentially expressed genes among all congenics was far greater than the number of eQTLs and that of differentially expressed genes between parental GK and BN strains (Figure 7). Even though animals were thoroughly genotyped throughout the backcross breeding and inbreeding stages required for the production of congenics, residual allele contaminants and gene conversions may have remained undetected and may account for abundant gene differential expression in congenics. Concordant transcriptional regulation between congenics, F2, and parental GK and BN strains was proportional (25.9–38.4%) to the fraction of the overall gene density and genomic length of the introgressed regions in the congenic intervals, indicating that *trans*-mediated mechanisms of gene expression can be efficiently uncovered in congenics. Comparison of transcription patterns in all three experimental systems demonstrated consistent allelic effects on gene expression for a subset of 232 genes (Figure 7). Transcription of a high proportion of these genes (83.6%) was regulated *in cis* by eQTLs in the cross, as defined by transcripts mapped within 10 Mb of markers showing the strongest evidence of linkage. However, 39.3% of these presumably *cis*-eQTL genes mapped outside congenic intervals (*i.e.*, F2 data suggested *cis*-regulation but congenics demonstrated *trans*-regulation), indicating additional contribution of *trans*-mediated control in the expression of these genes. eQTL reports in humans, suggesting that *trans* genetic variants can regulate multiple transcripts (Grundberg *et al.* 2012), support this observation.

We were able to validate only a fraction of *trans*-regulated eQTLs in congenics and conversely identified an important proportion of differentially expressed genes (83–89%) which were neither differentially expressed between GK and BN strains nor detected as eQTLs in the cross (Figure 7). Our findings suggest that genetic polymorphisms orchestrate gene expression regulation in different structures of gene × gene and gene × environment interactions when fixed homozygous in the genome or when segregating in an F2 cohort. However, due to statistical considerations of methods applied to eQTL mapping and gene differential expression analysis between groups of individuals, a larger F2 cohort would be required to test this hypothesis. Transcriptional changes in our congenic series are crude functional consequences of homozygous genetic variants from the donor strain present in each of the genomic blocks, interacting with specific collections of fixed alleles from the recipient strain in the genetic background. Variations in phenotypic features among congenics (Figure S4) may also explain incomplete concordance of transcriptome data in these experimental systems. Congenic data suggest that epistatic regulations and gene × environment interactions may have a much more prominent impact on gene transcription than expected by estimates derived from eQTL experiments performed in genetically heterogeneous cohorts.

At pathway level, transcriptome data in the GK × BN F2 cross identified changes in inflammatory and metabolic mechanisms, which are relevant to diabetes pathogenesis and are caused by genome-wide segregation of genetic polymorphisms. In contrast, each congenic transcriptome provided biological signatures of a subset of linked genetic polymorphisms in a specific genomic interval, which can be only partly replicated in F2 hybrids. An important finding in the congenics was that distinct genetic polymorphisms in disjoint genomic blocks control the same biological pathways, suggesting functional redundancy of genetic control. This phenomenon, which was also reported in the budding yeast where about 15% of genes affect growth rate (Sopko *et al.* 2006), is proposed as a mechanism to ensure sustained maintenance of essential phenotypes. Our data also provided experimental support to the concept that mammalian syndromes can be caused by mutations at different loci in unrelated genes, which nevertheless share functional relationships with the disease phenotype (Brunner and van Driel 2004).

### Conclusions

Results from our transcriptome analyses demonstrate the power of congenic series to dissect out *cis*- and *trans*-mediated mechanisms of gene expression, to attach biological functions to linked polymorphisms in genomic blocks, and to validate eQTL hotspots. Our data underline the importance of systems genetics (Civelek and Lusis 2014) to enhance knowledge of fundamental mechanisms, including functional epistasis and gene functional redundancy, which contribute to modulating the function of disease susceptibility genes and affect pathophysiological aspects of complex disorders.

## Supplementary Material

Supplemental Material
