# Supplementary material for: Transcriptome Profiling in Rat Inbred Strains and Experimental Cross Reveals Discrepant Genetic Architecture of Genome-Wide Gene Expression
Source: G3 (Bethesda). 2016 Sep 19;6(11):3671–83. doi: 10.1534/g3.116.033274 (PMC5100866; doi:10.1534/g3.116.033274)
Supplement: Supplemental Material [file supp_6_11_3671__index.html]

Transcriptome Profiling in Rat Inbred Strains and Experimental Cross Reveals Discrepant Genetic Architecture of Genome-Wide Gene Expression — Supplemental Material 

# Transcriptome Profiling in Rat Inbred Strains and Experimental Cross Reveals Discrepant Genetic Architecture of Genome-Wide Gene Expression

## Supplemental Material for Kaisaki, *et al*, 2016

**Files in this Data Supplement:**

- Table S1 - Genomic details of the BN.GK and GK.BN congenic strains. (.pdf, 106 KB)
- Table S9 - Overview of adipose tissue eQTLs detected in GKxBN F2 hybrids. (.pdf, 8 KB)
- Figure S1 - Validation of selected eQTLs by qRTPCR in adipose tissue of relevant SN.GK congenic strains (A) and transcription analysis of genes containing sequence variants between GK and BN in illumina oligonucleotides (B). (.tif, 1,226 KB)
- Figure S2 - Effects of introgressed haplotypes in congenic strains on trans-regulated fat gene transcription. (.tif, 5,533 KB)
- Figure S3 - Correlation analysis of the effects of GK alleles on the genetic control of gene expression in F2 hybrids and congenic strains. (.tif, 140 KB)
- Figure S4 - Phenotype analyses in BN.GK congenic strains. (.tif, 109 KB)
- Table S2 - Identification and chromosomal location of Illumina oligonucleotides carrying polymorphisms between the GK strain and the BN reference sequence. (.xlsx, 42 KB)
- Table S3 - Oligonucleotides used for eQTL validation by quantitative RT PCR in congenic strains and GK and BN parental strains. (.xlsx, 24 KB)
- Table S4 - Details of genes significantly differentially expressed (adjusted P<0.05) in adipose tissue between GK and BN strains and between BN.GK and GK.BN congenic strains and relevant BN or GK controls, respectively, and eQTL data in the GKxBN F2 cross. (.xlsx, 4790 KB)
- Table S5 - Effects of fat gene expression changes between congenics and controls on KEGG biological pathways. (.xlsx, 14 KB)
- Table S6 - Effects of fat gene expression changes between congenics and controls on Reactome biological pathways. (.xlsx, 14 KB)
- Table S7 - Genes contributing to enrichment of KEGG pathways in BN.GK congenics sharing GK genomic blocks and in reciprocal congenic strains (BN.GK and GK.BN) shown in Figure 4B-E. (.xlsx, 23 KB)
- Table S8 - Genes contributing to enrichment of the ribosome pathways in BN. (.xlsx, 22 KB)
- Table S10 - Details of the adipose tissue eQTL hotspot detected on chromosome 1 (49.4-59.4cM) in the GKxBN F2 cross and expression pattern of the linked gene in the congenic strain BN.GK1p. (.xlsx, 85 KB)
