# Supplementary material for: Transcriptome Profiling in Rat Inbred Strains and Experimental Cross Reveals Discrepant Genetic Architecture of Genome-Wide Gene Expression
Source: G3 (Bethesda). 2016 Sep 19;6(11):3671–83. doi: 10.1534/g3.116.033274 (PMC5100866; doi:10.1534/g3.116.033274)
Supplement: Supplemental Material [file supp_g3.116.033274_TableS1.pdf]

Table S1. Genomic details of the BN.GK and GK.BN congenic strains. Name and genomic position (Mb) of the genetic markers flanking the GK (BN.GK congenics) (a) or BN (GK.BN congenics) (b) genomic blocks are given, alongwith the minimum and maximum genomic length (Mb) of the introgressed GK or BN genomic segments and the approximate gene content. Data are based on ENSEMBL rat genome annotations (RGSC3.4, Ensembl release 69).

Chr	a- BN.GK congenic name	Congenic block	Gene density	Flanking background marker	First congenic marker	Last congenic marker	Flanking background marker
		length (Mb)		BN allele	GK allele	GK allele	BN allele
1	BN.GK-1b	20.2-29.9	203	D1Rat77 (238.0)	D1Got237 (246.7)	D1Got353 (266.9)	- (Telomere)
1	BN.GK-1d	3.7-7.2	24	J576143 (225.8)	J337594 (227.9)	D1Got231 (231.6)	D1Got224 (233.0)
1	BN.GK-1f	39.0-42.1	394	J576143 (225.8)	J337594 (227.9)	D1Got353 (266.9)	- (Telomere)
1	BN.GK-1h	62.34-70.3	994	D1Got337 (193.5)	D1Got338 (197.0)	D1Rat84 (259.3)	D1Cebr4 (263.8)
1	BN.GK-1p	99.2-103.1	1407	D1Got96 (88.6)	D1Rat27 (90.3)	D1Smu5 (189.5)	D1Got172 (191.7)
1	BN.GK-1q	9.3-11.8	90	D1Wox86 (224.7)	J576143 (225.8)	D1Rat75 (235.1)	D1Wox89 (236.5)
1	BN.GK-1t	0.9-4.6	9	J576143 (225.8)	J337594 (227.9)	D1Rat223 (228.8)	D1Rat76 (230.4)
1	BN.GK-1u	31.6-37.6	541	D1Wox7 (139.0)	D1Wox78 (143.8)	D1Got191 (175.4)	D1Got326 (176.6)
1	BN.GK-1v	24.9-35.5	210	D1Wox18 (94.6)	D1Got307 (102.5)	D1Got108 (127.4)	D1Got318 (130.1)
2	BN.GK-2c	144.7- 183.0	1243	D2Wox30 (46.3)	D2Mit6 (77.6)	D2Got149 (222.3)	D2wox68 (229.3)
4	BN.GK-4b	144.8-169.5	1578	D4Mgh1 (17.6)	D4Mgh14 (36.6)	D4Mgh13 (181.4)	- (Telomere)
5	BN.GK-5a	64.8-80.7	511	D5Got220 (43.3)	D5Mgh5 (45.5)	D5Wox24 (110.3)	D5Mit6 (124.0)
5	BN.GK-5c	43.2-45.5	259	- (Centromere)	D5Got201 (0.1)	D5Got220 (43.3)	D5Mgh5 (45.5)
7	BN.GK-7a	39.8-42.5	576	D7Wox42 (96.5)	D7Wox14 (99.0)	D7Cebr1 (138.8)	- (Telomere)
7	BN.GK-7d	30.7-34.4	275	D7Got54 (84.9)	D7Mit5 (85.1)	D7Wox25 (115.8)	D7Got104 (119.3)
8	BN.GK-8b	38.9-51.9	448	D8Got49 (38.9)	D8Got302 (48.2)	D8Got130 (87.1)	D8Got308 (90.8)
10	BN.GK-10a	66.5-84.7	1432	D10Mgh12 (19.1)	D10Wox26 (30.3)	D10Mgh4 (96.8)	D10Got156 (103.8)
	b- GK.BN congenic name			GK allele	BN allele	BN allele	GK allele
7	GK.BN-7a	104.8-111.8	1014	D7Got19 (31.2)	D7Mit7 (34.0)	D7Cebr1 (138.8)	- (Telomere)
8	GK.BN-8a	94.1-108.1	1149	- (Centromere)	D8Mit6 (9.6)	D8Wox6 (103.7)	D8Wox25 (108.1)
7 + 10	GK.BN-10_7a	158.0- 183.7	2166	D7Got19 (31.2)	D7Mit7 (34.0)	D7Cebr1 (138.8)	- (Telomere)
				D10Wox49 (37.7)	D10wox12 (56.4)	D10Got221 (109.6)	- (Telomere)
