# Supplementary material for: Transcriptome Profiling in Rat Inbred Strains and Experimental Cross Reveals Discrepant Genetic Architecture of Genome-Wide Gene Expression
Source: G3 (Bethesda). 2016 Sep 19;6(11):3671–83. doi: 10.1534/g3.116.033274 (PMC5100866; doi:10.1534/g3.116.033274)
Supplement: Supplemental Material [file supp_g3.116.033274_TableS9.pdf]

Table S9. Overview of adipose tissue eQTLs detected in GKxBN F2 hybrids. Chromosome length and number of protein-coding genes are based on ENSEMBL annotations of the rat genome (RGSC3.4, Ensembl release 69). Statistical models were applied to map eQTL effects using sex and cross as additive covariates (additive model). The total number of eQTLs detected is given for each chromosome and the number of eQTLs mapped within 10Mb of the linked transcript, which putatively correspond to cis-regulated effects, is reported in parentheses. Details of eQTLs and relationships with differentially expressed genes in congenics are given in S8 Table.

Chr	Length (Mb)	Genes	N eQTLs
1	267.9	3030	740 (112)
2	258.2	1488	116 (67)
3	171.1	1810	114 (59)
4	187.1	1501	145 (56)
5	173.1	1405	165 (69)
6	147.6	960	92 (43)
7	143.0	1390	208 (56)
8	129.0	1241	120 (60)
9	113.4	762	96 (28)
10	110.7	1722	182 (84)
11	87.8	615	39 (18)
12	46.8	606	60 (35)
13	111.2	699	125 (26)
14	112.2	796	69 (25)
15	109.8	818	55 (17)
16	90.2	653	39 (24)
17	97.3	700	116 (36)
18	87.3	550	41 (23)
19	59.2	585	77 (25)
20	55.3	621	66 (45)
X	160.7	1028	70 (2)
Total	2718.9	22980	2735 (910)
