# Supplementary figures and images for: Transcriptome Profiling in Rat Inbred Strains and Experimental Cross Reveals Discrepant Genetic Architecture of Genome-Wide Gene Expression
Source: G3 (Bethesda). 2016 Sep 19;6(11):3671–83. doi: 10.1534/g3.116.033274 (PMC5100866; doi:10.1534/g3.116.033274)

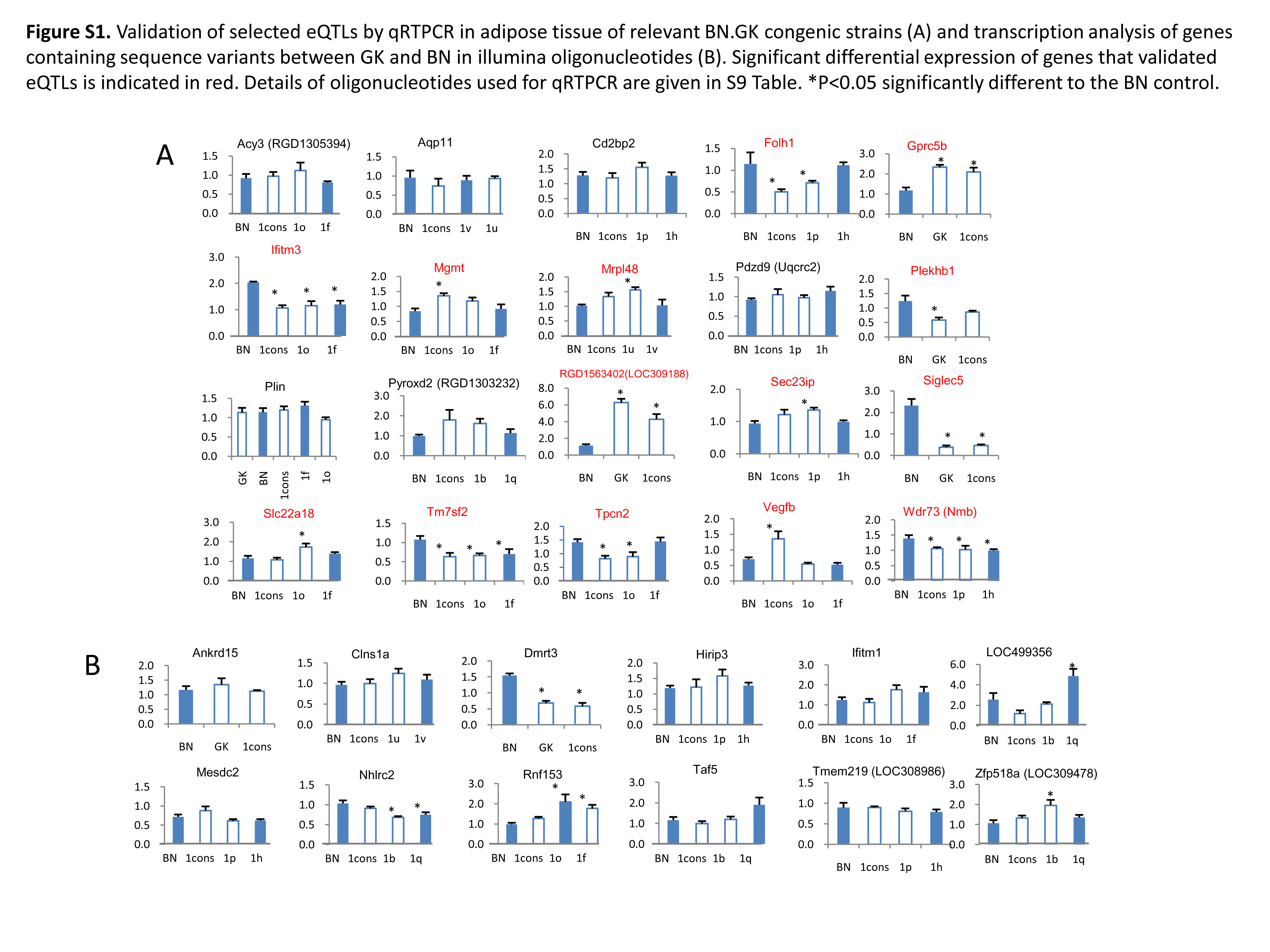

Supplement: Supplemental Material [file supp_g3.116.033274_FigureS1.tif]

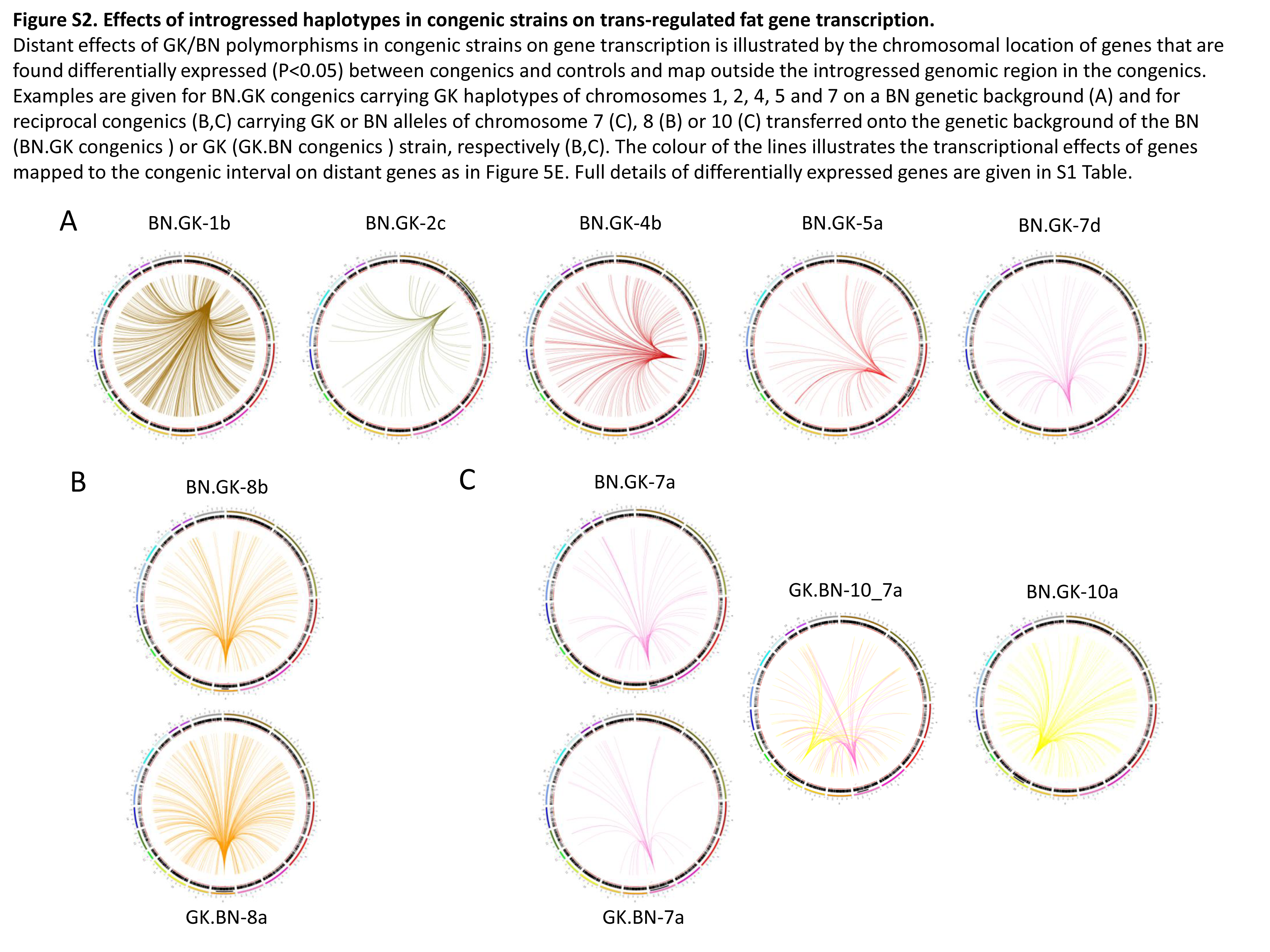

Supplement: Supplemental Material [file supp_g3.116.033274_FigureS2.tif]

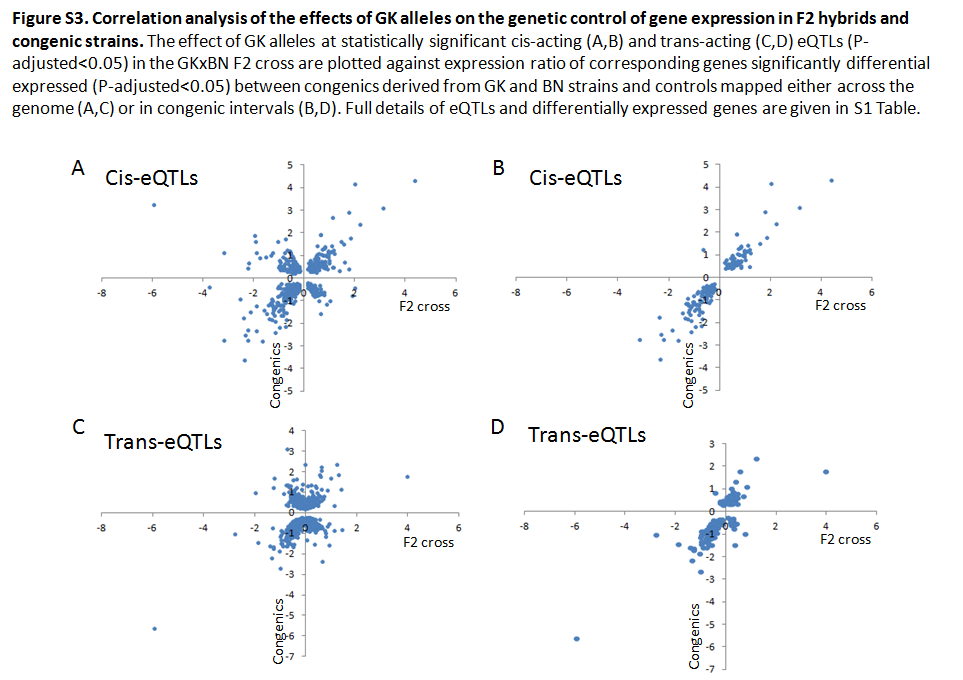

Supplement: Supplemental Material [file supp_g3.116.033274_FigureS3.tif]

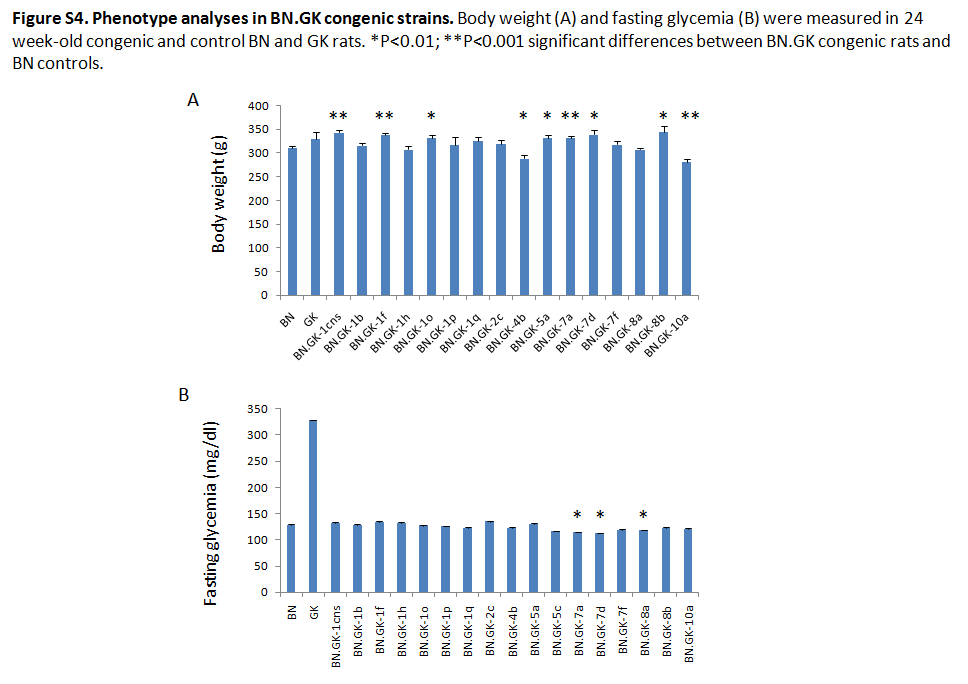

Supplement: Supplemental Material [file supp_g3.116.033274_FigureS4.tif]
